# Design and Development of Cellulosic Bionanocomposites from Forestry Waste Residues for 3D Printing Applications

**DOI:** 10.3390/ma14133462

**Published:** 2021-06-22

**Authors:** Maya Jacob John, Nokuzola Dyanti, Teboho Mokhena, Victor Agbakoba, Bruce Sithole

**Affiliations:** 1 Centre for Nanostructures and Advanced Materials, Council for Scientific and Industrial Research (CSIR), Pretoria P.O. Box 395, South Africa; dyanti@gmail.com (N.D.); TMokhena@csir.co.za (T.M.); s215161440@mandela.ac.za (V.A.); 2 Department of Chemistry, Nelson Mandela University, Port Elizabeth P.O. Box 77000, South Africa; 3 Biorefinery Industry Development Facility (BIDF), Council for Scientific and Industrial Research (CSIR), Durban P.O. Box 59081, South Africa; bsithole@csir.co.za

**Keywords:** cellulose nanofibres, biopolymer, bionanocomposites, 3D printing

## Abstract

This paper deals with the development of cellulose nanofibres (CNFs) reinforced biopolymers for use in packaging applications. Cellulose nanofibres were extracted from sawdust by a combination of chemical and mechanical treatments. The extracted cellulose nanofibres were chemically modified (fCNFs) and characterised by Fourier Transform Infrared Spectroscopy (FTIR). Bionanocomposites were prepared from biopolymers polylactic acid/polybutylene succinate (PLA/PBS) and cellulose nanofibres by compounding in a twin-screw extruder followed by injection moulding. The developed bionanocomposites were subjected to mechanical and thermal characterisation. As part of product development, CNF-biopolymer pellets were also extruded into filaments which were then 3D printed into prototypes. This work is a successful demonstration of conversion of waste residues into value-added products, which is aligned to the principles of circular economy and sustainable development.

## 1. Introduction

Currently, there is a trend towards the use of sustainable packaging materials due to increasing environmental legislations regulating the biobased content in packaging products. South African packaging materials available in the market and used for fruit export are derived from petroleum-based polymers (polypropylene) and there is a demand to change to packaging materials (crates, cartons and retail bags) with lower carbon footprint by using bio-based materials and/or energy efficient processing techniques. This also forms part of the circular economy and a new plastics vision roadmap for plastics packaging which stresses the elimination of plastics that cannot be recycled or composted, innovation for development of new compostable plastics and circulation of plastic products within the economy so that plastics do not end up in the environment [1].

Amongst biopolymers, polylactic acid (PLA) has favourable properties such as excellent mechanical strength, being industrially compostable and having easy processability on conventional processing equipment (extrusion and compression/injection moulding). Additionally, PLA offers a significantly reduced carbon footprint (approximately 70% reduction) when compared to oil-based plastics and requires less energy for processing compared to other biobased non-biodegradable plastics such as polyolefins. It is, however, recognised that PLA is a brittle polymer, which limits its success in various packaging, biomedical and pharmaceutical applications. Therefore, in order to extend its applicability in various fields, the addition of bio-based reinforcements such as cellulose nanofibres and/or blending with more ductile/flexible biopolymers serve as a suitable solution because they do not compromise its biodegradability. The blending of PLA with polybutylene succinate (PBS) [2] is gaining traction as PBS is biodegradable and has favourable melt processability and strength properties comparable to conventional polymers such as polyethylene and polypropylene. However, PLA/PBS blends are immiscible [3] and would require compatibilisers to increase interfacial adhesion and obtain materials with superior properties. In this context, the use of cellulose nanomaterials (cellulose nanofibres and cellulose nanocrystals) as a bio-based reinforcing [4,5] agents (due to high strength and stiffness values) and to improve compatibility of immiscible systems is being explored [6]. Additionally, cellulose nanomaterials can be extracted from various waste biomass residues [7], paving the way for conversion of waste into high value-added products [8]. One of the problems associated with cellulose nanofibres (CNFs) is the dispersion and compatibility in biopolymer matrices, which are usually remedied by the use of chemical treatments. Although there are studies on the reinforcement of CNFs [9,10] and other types of fillers in biopolymer blends [11,12], reports on the use of environmentally friendly modified CNFs and its effect on the properties of the biopolymer blends is limited.

In this work, cellulose nanofibres were extracted from sawdust by a combination of chemical and mechanical treatments. The extracted cellulose nanofibres were subjected to bio-based modification using canola oil and characterised using FTIR. Bionanocomposites were developed from untreated and treated cellulose nanofibres and PLA/PBS blends. The mechanical and thermal characteristics of the developed samples (PLA/PBS blends, CNFs-PLA/PBS and fCNFs-PLA/PBS) were evaluated. As part of product development, cellulose nanofibres reinforced PLA were extruded to form filaments and then 3D printed to form packaging crates.

## 2. Materials

Biopolymers—cPLA 1001 (Cereplast Inc, Hawthorne, CA, USA) resin with melt flow index (MFI) of 4.3 g/10 min—was purchased from Cereplast (Hawthorne, NY, USA). PBS (Bionolle^TM^ 3001) with the melt flow index and density of 3 g/10 min and 1.23 g/cm^3^, respectively, was purchased from SHOWA Highpolymer Co. Ltd. (Tokyo, Japan). Black PLA filament (CRON) was procured for 3D printing.

Chemicals for extraction of cellulose nanofibres and chemical modification—NaOH (99.5%), KOH (99.5%), K_2_CO_3_ (99.5%), hexane (99.9%) and ethanol (99.9%)—were procured from Ibhayi Laboratory Supplies (Port Elizabeth, South Africa). All chemicals were used as received without purification. Canola oil (B-Well, Swellendam, South Africa) was obtained from a local supermarket. Eucalyptus sawdust (used for the production of cellulose nanofibres) was supplied by the Council for Scientific and Industrial Research (CSIR) Biorefinery Development Facility (BIDF) in Durban, South Africa.

## 3. Experimental

### 3.1. Extraction of Cellulose Nanofibres (CNFs) from Saw Dust

**Figure 1:**
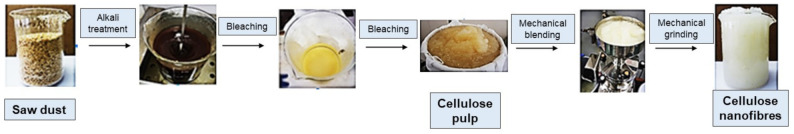
Extraction of cellulose nanofibres (CNFs) from eucalyptus saw dust.

Saw dust powder was subjected to chemical treatment comprising 1% NaOH and bleaching agent (NaHClO_3_) under optimised temperature (80°) and time profiles (4–6 h). The cellulose pulp obtained was filtered and washed with deionised water until neutral pH was achieved. The obtained white powder was subjected to mechanical grinding using a Supermass collider (MKCA6-39Masuko Sangyo Co, Ltd., Saitama, Japan) at 1500 rpm for 20 min until a gel-like substance was obtained (Figure 1).

### 3.2. Chemical Modification of Cellulose Nanofibres

**Scheme 1:**
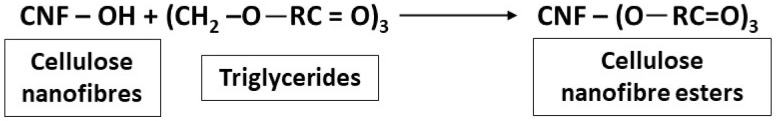
Reaction between cellulose nanofibres and triglycerides.

Canola oil was used for surface modification of the CNFs resulting in a transesterification reaction between hydroxyl groups of the CNFs and fatty acid groups in the triglycerides in canola oil [13] (Scheme 1). A suspension containing CNFs was solvent-exchanged to ethanol. This process was performed four times by centrifugation at 10 °C and 10,000 rpm for 15 min. After each step, re-dispersion of the suspension was carried out using a mechanical blender. A pre-weighed amount of canola oil was added to 100 mL of CNF suspension in ethanol and further subjected to blending for 10 min. The treated samples were dried at room temperature in order to ensure the complete removal of ethanol. After drying at room temperature, the samples were kept in an oven (110 °C) for 2 h. The resulting samples were further washed with ethanol to remove unreacted canola oil and then dried at room temperature. The chemically modified CNFs were represented as fCNFs.

### 3.3. Preparation of cPLA/PBS Biopolymer Blends

#### 3.3.1. Processing of Blends

Biopolymer blends were developed by melt compounding process in a twin-screw extruder followed by injection moulding to form the dumbbell-shaped specimens for testing.

#### 3.3.2. Preparation of Blends by Injection Moulding

A twin-screw extruder equipped with a feeder and a strand pelletizer was used to compound the blends. PLA and PBS pellets were dried in a convection oven at 60 °C for 8 h before extrusion. The extrusion temperature profile was set from 185 to 200 °C along the barrel and the screw speed was maintained at 45 rpm for all samples. Samples containing varying loadings of PBS and PLA were prepared. After exiting the die, the extrudate was cooled in a water bath before being granulated by a pelletizer. The compounded pellets were dried in an oven at 60 °C for 8 h and then injection moulded at a nozzle temperature of 200 °C to obtain the dumbbell-shaped specimens for mechanical testing.

#### 3.3.3. Preparation of Bionanocomposites from CNF’s (Treated and Untreated) and PLA/PBS Blends

The blend 70/30 PLA/PBS exhibited higher strain values for rigid packaging applications, and thus this ratio was chosen for the incorporation of untreated and chemically modified cellulose nanofibres. The masterbatch (containing cPLA/CNFs and cPLA/fCNFs) were mixed in a twin-screw extruder to obtain pellets, which were injection moulded to form testing specimens.

### 3.4. Characterization

Tensile and three-point bending tests were carried out using an Instron Universal Testing Machine, model 3369 (Instron, Norwood, Massachusetts)manufacturer, city, state, USA). Tensile testing on dumbbell specimens (165 mm × 19 mm × 3 mm) was measured according to ASTM methods D368 at a crosshead speed of 50 mm/min and a gage length of 50 mm. Flexural testing was carried out rectangular specimens (130 mm × 12.7 mm × 3 mm) in accordance with ASTM D-790, at a crosshead speed of 5 mm/min and a span length of 60 mm. Five specimens were tested and the average data have been reported.

Differential scanning calorimetry (DSC) was carried out with a Diamond DSC (PerkinElmer, Waltham, MA, USA) on samples of 5–10 mg. An empty pan was used as reference. Samples were scanned from 30 to 200 °C at a heating rate of 10 °C/min under a nitrogen flow rate of 20 mL/min, kept at this temperature for 2 min to erase the thermal history, cooled to 30 °C at the same rate and then reheated under the same conditions. Three repeat measurements were performed to ensure reproducibility.

Thermogravimetric analysis (TGA) studies were carried out using a Pyris 1 model (PerkinElmer, Waltham, MA, USA) in an inert atmosphere at a heating rate of 10 °C/min. Samples with masses in the range of 5–10 mg were scanned from 30 to 700 °C. Three specimens were tested and the average data have been reported.

Fourier Transform Infrared spectra of cellulosic nanofibres and chemically modified nanofibres were recorded with an FT-IR spectrometer (PerkinElmer, Waltham, MA, USA) Spectrum 100 FTIR Spectrometer with fitted ATR (Attenuated Total Reflectance) sampling accessory). The spectra were recorded using 32 scans and a resolution of 4 cm^−1^ over a range of 650–4000 cm^−1^.

Transmission electron microscopy (TEM) studies of the extracted CNFs were performed on TEM CM 200 equipment (Philips, Amsterdam Netherlands). A suspension diluted with water to 0.01% w/v was deposited on a carbon grid, stained with 2% of uranyl acetate and allowed to dry at room temperature prior imaging. The grids were observed at an accelerating voltage of 80 kV.

## 4. Results and Discussion

### 4.1. Characterization of Chemically Modified Cellulose Nanofibres

#### 4.1.1. Fourier Transform InfraRed Spectrometer (FTIR) Studies

**Figure 2:**
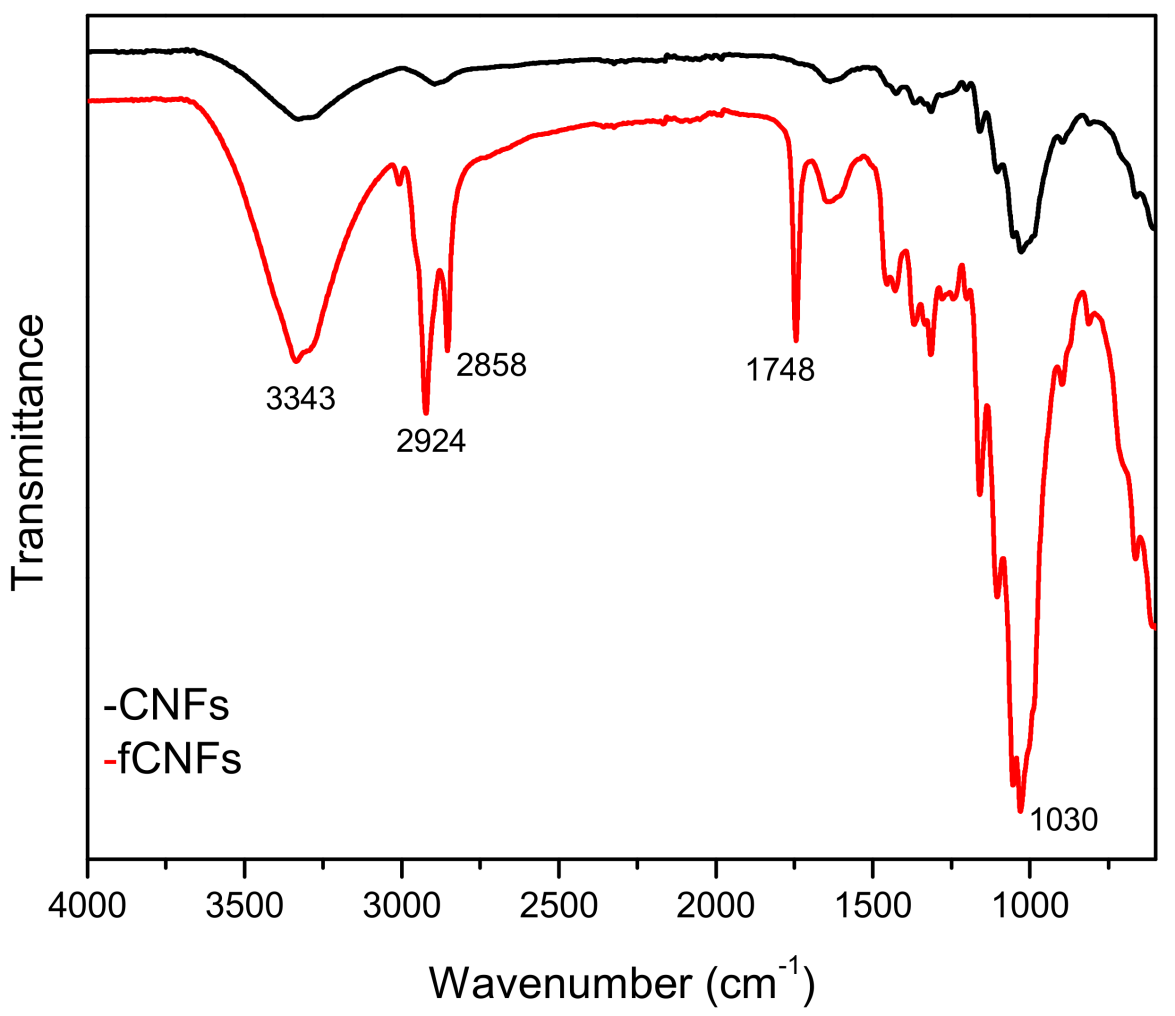
FTIR spectra for cellulose nanofibres (CNFs) and functionalised cellulose nanofibres (fCNFs).

FTIR spectra for CNFs and functionalised CNFs (fCNFS) are shown in Figure 2. The typical characteristic vibrations of cellulose at 3343–3315 cm^−1^ corresponding to –OH groups in cellulose are seen in both samples [14]. In fCNFs, the absorption bands at 2858 and 2924 cm^−1^ for methyl and methylene stretching became more intense, suggesting that esterification reactions have occurred. The peak at 1636 cm^−1^ in CNFs is attributed to the presence of H–O–H and this has become more intense in fCNFs, signifying that the hydroxyl groups have reacted with the acid groups in canola oil. Additionally, a new strong absorption peak at 1748 cm^−1^ associated with carbonyl stretching of aliphatic esters was observed for functionalised CNFs, indicating that esterification reaction was successful [15]. No absorption band was observed at about 1730 cm^−1^ for either CNFs or fCNFs, which corresponds to C=O stretching vibrations of the acetyl and uronic ester groups of hemicellulose, suggesting that hemicellulose was successfully removed via chemical and mechanical treatments. The bands around 1030, 1090  and 1158 cm^−1^ present in the spectra of the CNFs are more intense in the case of fCNFs and are attributed to C=O stretching, C–O–C asymmetric stretching and C–H oscillating vibrations of cellulose, respectively [16]. The peak at 897 cm^−1^ represents the typical characteristics of cellulose structure and is attributed to the β-glycosidic bond of cellulose. Several studies on FTIR characterisation of cellulose and CNFs extracted by chemical and mechanical methods have reported similar observations [17,18]. The main absorption bands and characteristic functional groups are tabulated in Table 1.

#### 4.1.2. TEM Studies of CNFs

**Figure 3:**
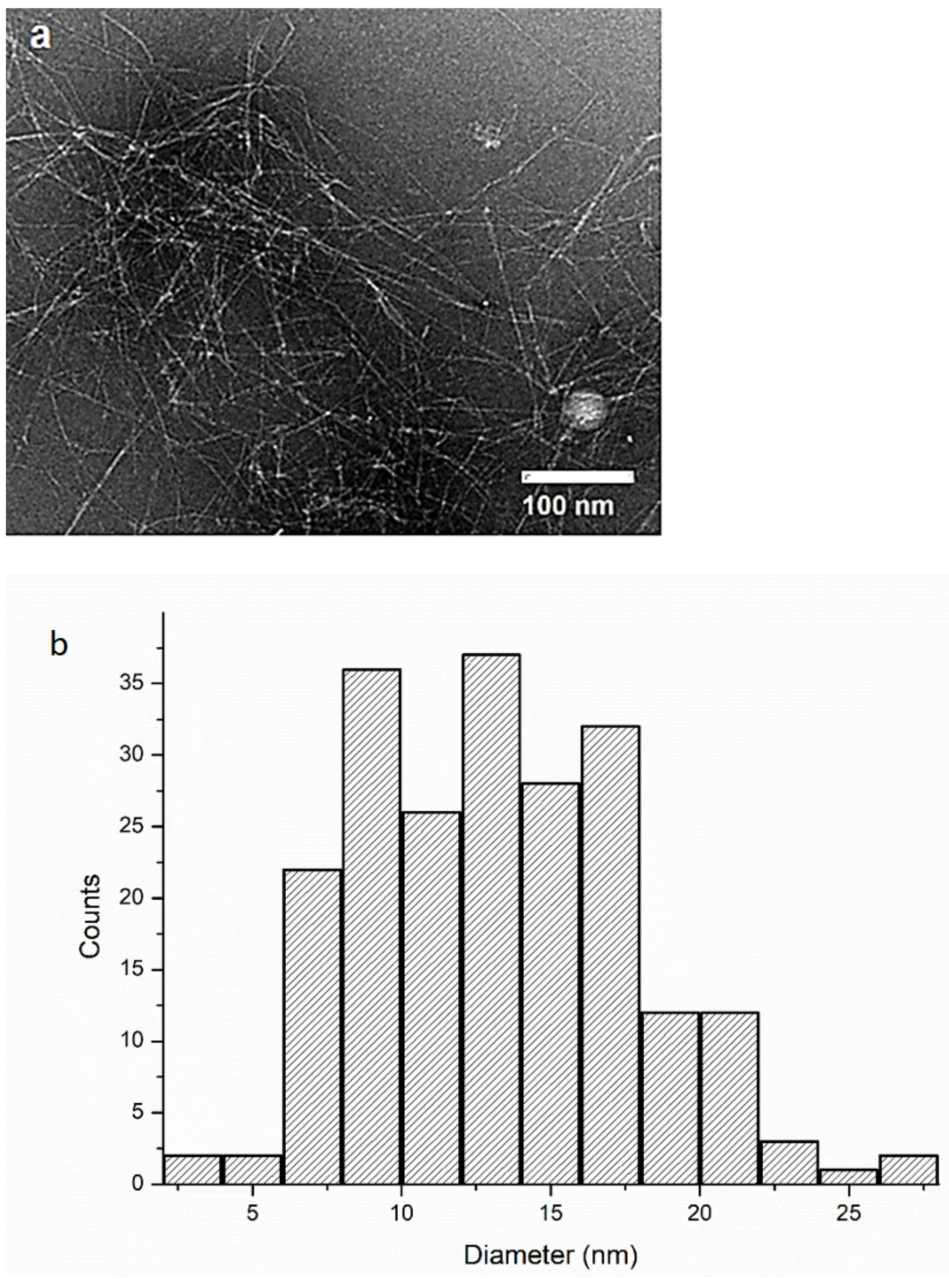
(**a**) Transmission electron microscopy (TEM) micrograph of CNFs and (**b**) with diameter distribution.

TEM studies were performed to analyse the sizes and morphology of the CNFs extracted from sawdust [13]. Figure 3a presents the TEM micrograph of the CNFs and it can be observed that the CNFs displayed a web-like structure. The pre-treatment of the sawdust with NaOH, bleach and KOH removed other constituents such as hemicellulose and lignin and mechanical grinding using Supermass collider facilitated the defibrillation of fibres, resulting in extracted CNFs having diameters ranging between 2 and 27 nm and lengths reaching few microns. The TEM images also reveal areas where the CNFs are aggregated close to the concentration gradients of the stained areas. Furthermore, in Figure 3b, it can be observed that the CNFs had a narrow size distribution, with most of the nanofibres having diameter between 8 and 16 nm based on 200 measurements averaged from all the TEM images. Similar results on diameters have been reported by researchers regarding extraction of cellulose nanofibres from hemp fibres (20–50 nm) [23] and sugar palm (21.37 nm) [24].

### 4.2. Mechanical Properties of the Biopolymer Blends

Table 2 presents the tensile properties of the biopolymer blends. PLA exhibited higher tensile strength (41.37 MPa) and modulus (2204.54 MPa) when compared to PBS. As expected, the incorporation of PBS led to reduction in tensile strength and modulus with an increase in elongation at break values. At 30% PBS loading, the tensile strength and modulus decreased by 18% and 31%, respectively, while the elongation at break values increased by 20%. This is attributed to the mixing of a ductile flexible biopolymer with a rigid brittle biopolymer. The type of bonding between the biopolymer phases in a blend will influence the mechanical properties of the material. In this case, PLA and PBS form immiscible phases (seen in DSC studies), with PLA forming the continuous phase and PBS forming the dispersed phase. When the samples are subjected to tensile stress, the dispersed phases of soft PBS can act as stress concentrators, resulting in high stress pockets in the PBS domains and leading to debonding at the blend interface. Similar mechanical results have been reported by Bhatia et al. [25], who observed that blends with high content of PBS (>30%) showed a ductile behaviour with the occurrence of necking, while pure PLA and blends with low PBS content (<30%) showed a brittle fracture nature, with no sign of necking phenomenon. These results reveal that the brittle nature of pure PLA can be improved by blending with the ductile PBS biopolymer. Flexural properties also followed the same trend as reduction in flexural strength and modulus with increasing PBS content (Table 3). At 30% PBS loading, the flexural strength and modulus decreased by 20% and 30%, respectively.

**Figure 4:**
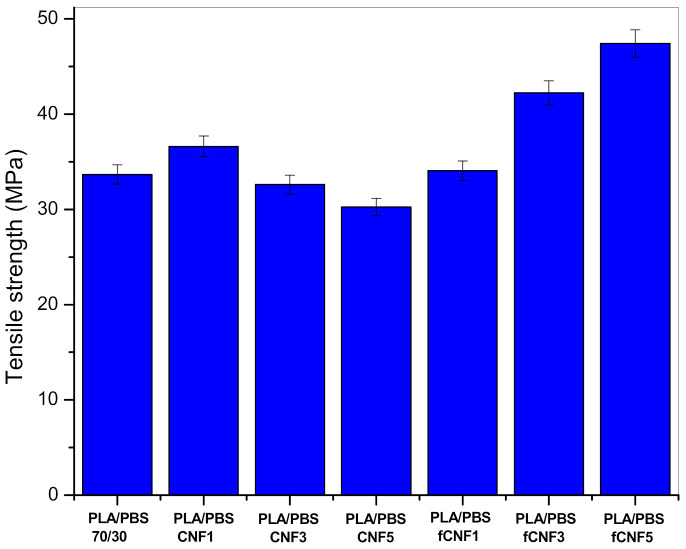
Tensile strength of bionanocomposites.

**Figure 5:**
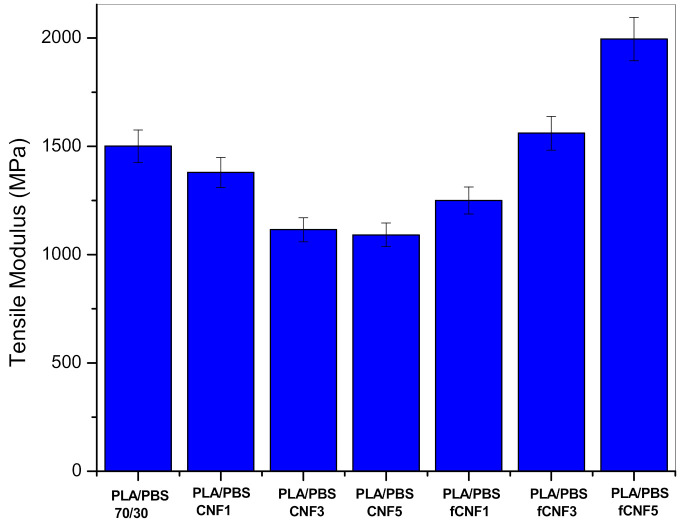
Tensile modulus of bionanocomposites.

Figure 4 and Figure 5 present the tensile results of the bionanocomposites containing untreated and chemically treated cellulose nanofibres. It can be observed that upon addition of cellulose nanofibres (1%), the tensile strength increases, and at higher loadings of CNFs (5%), decreases. This is attributed to the possible agglomeration of cellulose nanofibres at high loadings preventing effective transfer between the biopolymers and the cellulose reinforcement. The modulus and elongation at break values also register a decrease. In the case of bionanocomposites containing chemically modified CNFs, there is an improvement in both tensile strength and modulus by 40% and 30%, respectively, due to greater interfacial adhesion between biopolymers and functional groups in the CNFs. The functionalisation of cellulose nanofibres imparts hydrophobic characteristics due to the presence of long-chain hydrocarbon of the fatty acids. This allows for better dispersion of CNFs and the possibility of H-bonding between the ester groups of CNFs and biopolymers, leading to greater stress transfer and improved tensile properties. Wang et al. have reported similar results with an increment of 10% in tensile values in the case of cellulose nanofibre reinforced PLA-PBS blend system compatibilised with bis(1-(tert-butylperoxy)–1-methylethyl)–benzene when compared to uncompatibilised samples^9^.

### 4.3. Thermal Studies of Blends and Bionanocomposites

#### DSC Results

Table 4 presents the DSC (T_m_, T_g_, ΔH_m_, X_c(PLA),_ T_cc_ and ΔH_cc_) data of the PLA/PBS blend and nanocomposite samples. It can be observed that the melting temperatures of PLA and PBS are 150.1 °C and 112.5 °C, respectively. In the case of the blends, two melting temperatures are observed for all the blends ratios, indicating that the biopolymers form an immiscible system and crystallise individually. The melting temperature of the biopolymers in the blend do not show any significant trend; however, the enthalpy of fusion is seen to increase with PBS loading. The cold crystallisation temperature of PLA is observed at 100 °C and is seen to shift to lower temperatures at high PBS loading. The cold crystallisation of PBS is recorded at 99 °C and is difficult to observe in the blends due to overlap with the melting temperature of PBS. The crystallinity of PLA in the blends display a slight increment due to PBS acting as nucleating agents. The glass transition temperature of PLA is recorded at 59.4 °C and is seen to slightly increase with blending. In the case of bionanocomposites, the Tg and melting temperatures do not show any significant variation upon incorporation of CNFs and modified CNFs. The crystallinity values show an increase with the addition of the cellulose nanomaterials, supporting the fact that cellulosic nanomaterials can act as nucleating agents [26] and accelerate crystallisation behaviour. Similar observations have been reported by Luizi et al. [4], who observed that the addition of cellulose nanocrystals led to the increase in crystallinity of the blends.

**Figure 6:**
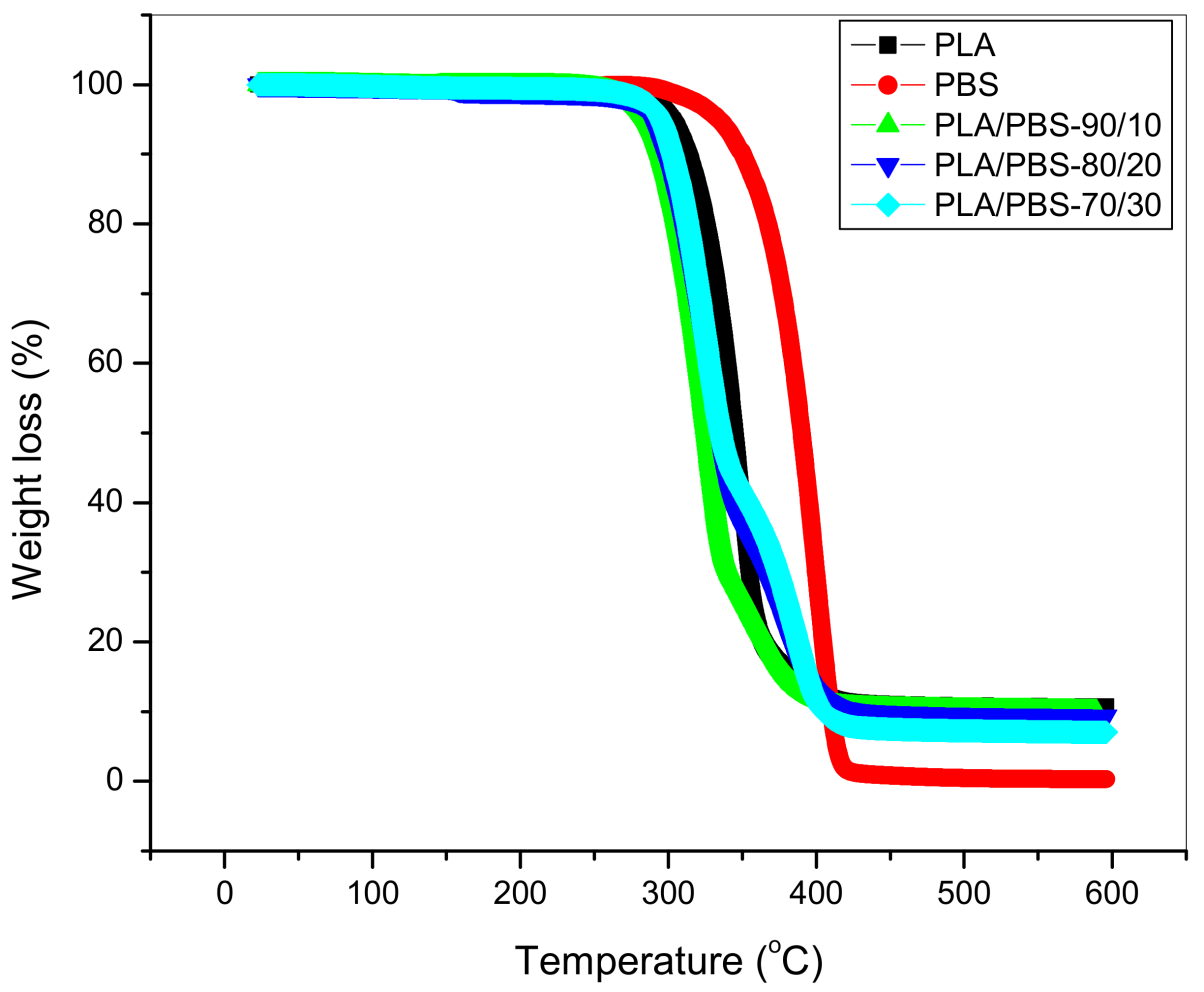
Thermograms of biopolymers and blends.

The thermal degradation behaviour of the blends is depicted in Figure 6. It can be observed that neat PLA and neat PBS exhibit a single step degradation profile with the onset temperature around 300 °C and 327 °C and a maximum temperature of thermal degradation at around 347 °C and 394 °C, respectively. Neat PBS undergoes a cyclic degradation mechanism—the prominent by-products are anhydrides, olefins, carbon dioxide and esters—and exhibits higher degradation temperature when compared to neat PLA [27]. In the case of the blends, two degradation peaks are observed at lower temperatures (345 °C) and (390 °C), which is associated with the degradation profiles of neat PLA and PBS, respectively. This is further evidence of a two-phase PLA/PBS blend system.

**Figure 7:**
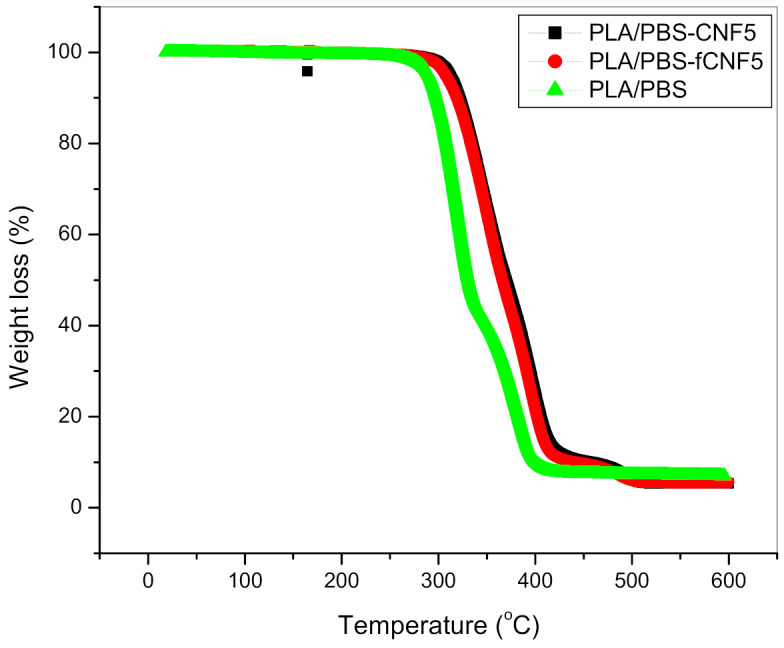
Thermograms of PLA/PBS blends and bionanocomposites.

In the case of the bionanocomposites, the thermal profiles of PLA/PBS and blends containing 5% functionalised and untreated CNFs are shown in Figure 7. It can be observed that samples containing CNFs are thermally more stable when compared to unreinforced blends and the maximum degradation temperatures have shifted to higher temperatures. This can be attributed to presence of CNFs acting as barriers to mobility of PLA/PBS macromolecular chains, resulting in delayed onset of degradation. Similar observations have been reported in the case of PLA composites containing acetylated microfibrillated cellulose (MFC), where the initial degradation temperature exhibited a 3% increase at 17% MFC loading [28].

### 4.4. Product Development Using 3-Dimensional (3D) Printing

Three-dimensional printing is an emerging technique that allows fabrication of a physical prototype using computer-aided design (CAD). The advantages of 3D printing when compared to conventional processing techniques such as extrusion and injection moulding are the following: design flexibility to produce customised products, expensive moulds are not required, less wastage and a much shorter time from design to production. Several 3D printing methods are available today, and they each require different forms of printing material (filament/resin/powder) and operate their own unique printing methods. The most widely used and cheapest technique today is the extrusion-based fused deposition modelling (FDM), in which thermoplastic filament is heated to melt, then extruded from the nozzle and deposited layer by layer on a support platform. For a polymer matrix to be used in FDM, there are several conditions such as: polymer melt should have proper viscosity and strength so that it can be consistently extruded out of the nozzle without breakage or buckling, volume shrinkage rate of polymer [29] must be minimum and the mechanical properties of the prototypes largely depend on the adhesion between the overlapping layers [30,31].

**Figure 8:**
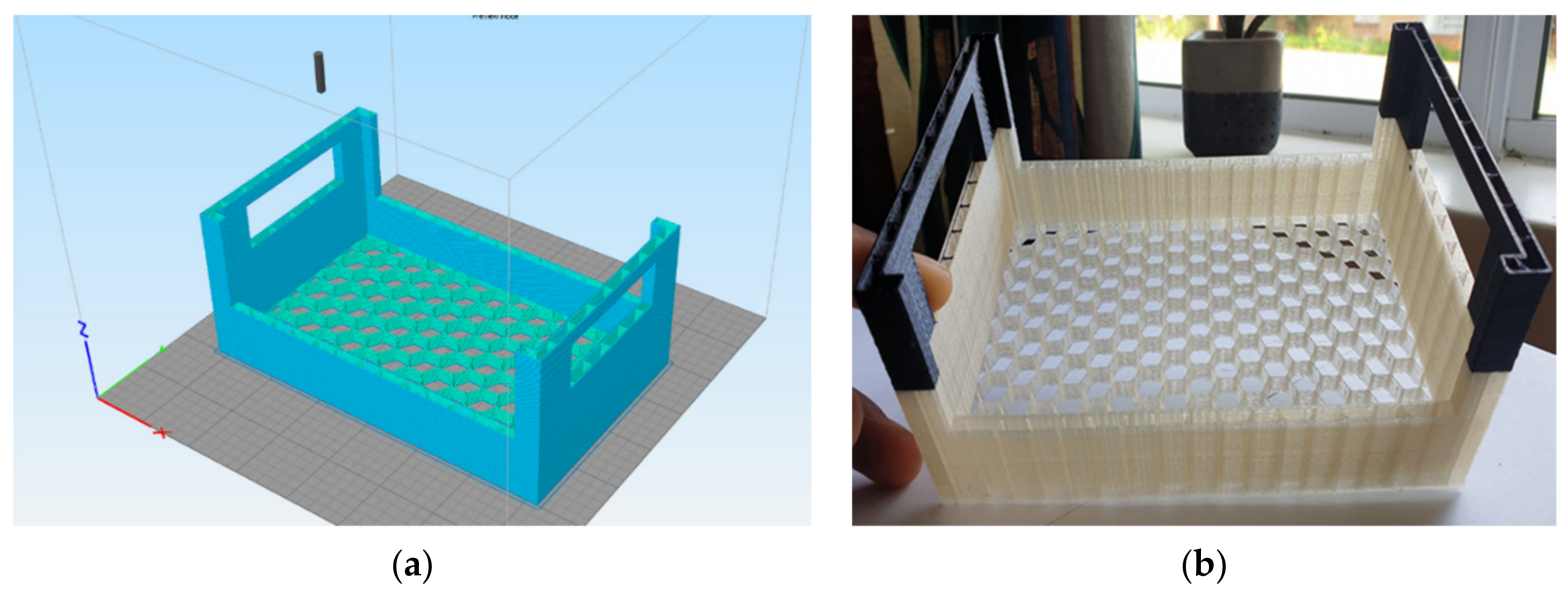
(**a**) Representation of the CAD model of crate after slicing; (**b**) 3D-printed crate prototype from PLA and cellulose nanofibres.

Figure 8a,b show the CAD model and the 3D printed prototype from PLA and cellulose nanofibres. The black handles of the prototype were printed using commercial grade black PLA filament while the main body was printed using the developed cellulose nanofibre reinforced PLA filaments. The computer-aided design (CAD) model of the crate was designed using Autodesk^®^ Tinkercad^®^ (Autodesk) software and saved as a stereolithography (STL) file. Three-dimensional printing was performed using a desktop 3D printer (Wanhao Duplicator i3 plus, Wanhao 3D Printer, Jinhua, China) fitted with a 0.4 mm nozzle. Simply3D^®^ slicing software was used to slice the crate’s STL file. Printing conditions were as follows: nozzle temperature 190 °C, bed temperature 50 °C, print speed of 60 mm/s, 2 perimeter wall, 0 top and bottom layers, primary layer height of 0.3 mm, 10% infill and full honeycomb infill design. A pause-resume function was performed at 75% print progress to switch from bionanocomposite filament to a commercial PLA.

## 5. Conclusions

Blends from PLA-PBS and bionanocomposites comprising unmodified (extracted from sawdust), esterified cellulose nanofibres and PLA-PBS blend matrix were successfully prepared by the process of melt compounding followed by injection moulding. The mechanical and thermal properties of the blends and bionanocomposites were analysed. It was observed that the flexibility of PLA was improved by the addition of ductile PBS. In the case of the bionanocomposites, mechanical properties were seen to decline upon the addition of cellulose nanofibres and this was attributed to the agglomeration of CNFs at the PLA/PBS interface. The incorporation of chemically modified CNFs improved the mechanical properties due to synergistic effect of presence of hydrophobic CNFs and improved bonding between CNFs and biopolymer blends. The thermal studies indicate that PLA and PBS form an immiscible system and the presence of CNFs improves the crystallinity of the blends due to nucleating effects. The thermal stability of the samples containing CNFs (untreated and chemically modified) was increased due to CNFs restricting the molecular motion in PLA/PBS chains. As part of prototype development, a crate was successfully 3D printed using CNFs reinforced PLA filaments. The work is a successful demonstration of sustainable conversion of waste into high performance bionanocomposites with tuneable properties for compostable packaging applications. Future studies will focus on microscopic studies to analyse the dispersion of CNFs with the biopolymer blend phases.

## Figures and Tables

**Table 1:** Major absorption bands and assigned chemical groups.

Wavenumber	Chemical Groups
3200–3300	–OH, Water [19]
2800–2900	–CH stretching vibration
1750	–C=O [20]
1600	–OH band of adsorbed water [21]
1000–1200	C–O stretching, C–O–C asymmetric stretching and C–H oscillating vibrations^,^ [13,14,22]

**Table 2:** Tensile properties of the biopolymer blends.

Specimen Label	Tensile Stress (MPa)	Tensile Modulus (MPa)	Tensile Strain (%)
PLA	41.37 ± 1.43	2204.45 ± 21.25	3.03 ± 0.11
PLA/PBS (90/10)	37.83 ± 0.91	1731.78 ± 35.74	3.53 ± 0.15
PLA/PBS (80/20)	36.31 ± 0.90	1603.63 ± 74.19	3.66 ± 0.15
PLA/PBS (70/30)	33.67 ± 0.96	1500.51 ± 88.09	3.67 ± 0.2
PBS	37.29 ± 2.01	287 ± 16.7	605 ± 62.19

**Table 3:** Flexural properties of the biopolymer blends.

Specimen Label	Flexural Stress (MPa)	Flexural Modulus (MPa)	Flexural Strain (%)
PLA	72.06 ± 1.38	3074.74 ± 132.58	4.18 ± 0.17
PLA/PBS (90/10)	65.62 ± 3.72	2709.37 ± 137.71	4.07 ± 0.17
PLA/PBS (80/20)	63.93 ± 0.68	2560.17 ± 51.89	4.15 ± 0.06
PLA/PBS (70/30)	57.11 ± 0.50	2129.92 ± 29.58	4.46 ± 0.08
PBS	30 ± 1.5	670 ± 80	250 ± 60.6

**Table 4:** Thermal parameters of virgin biopolymers, PLA/PBS blends and bionanocomposites.

Sample Description	Cold Crystallisation		Melt of Crystalline Region		Crystallinity	Glass Transition
	T_cc_ (°C)	ΔH_cc_ (J/g)	T_m_ (°C)	ΔH_m_ (J/g)	Χ_c(PLA)_ _(%)_	T_g_ (°C)
Neat PLA	100	22.7	150.1	28.5	6.2	59.4
Neat PBS	99	20.4	112.5	30.4	3.2	-
PLA/PBS (90/10)	98.3	23.0	149.4	29.6	8.5	60.0
			112.5			
PLA/PBS (80/20)	98.2	29.0	148.9	30.6	10.2	61.2
			111.6			
PLA/PBS (70/30)	98	30.1	148.8	33.7	13.5	61
			113.7			
PLA/PBS-CNF5	99	31.4	148.3	34.4	13.6	60.5
			112.4			
PLA/PBS-fCNF5	99.6	31.6	148.5	34.8	14.8	61
			112.9			

## Data Availability

Data is present within the article.
